# Genetic sequencing of a 1944 Rocky Mountain spotted fever vaccine

**DOI:** 10.1038/s41598-023-31894-0

**Published:** 2023-03-22

**Authors:** Yongli Xiao, Paul A. Beare, Sonja M. Best, David M. Morens, Marshall E. Bloom, Jeffery K. Taubenberger

**Affiliations:** 1 Viral Pathogenesis and Evolution Section, Laboratory of Infectious Diseases, National Institute of Allergy and Infectious Diseases, National Institutes of Health, 33 North Drive MSC 3203, Bethesda, MD 20892-3203 USA; 2 Coxiella Pathogenesis Section, Laboratory of Bacteriology, Rocky Mountain Laboratories, National Institute of Allergy and Infectious Diseases, National Institutes of Health, Hamilton, MT USA; 3 Innate Immunity and Pathogenesis Section, Laboratory of Persistent Viral Diseases, Rocky Mountain Laboratories, National Institute of Allergy and Infectious Diseases, National Institutes of Health, Hamilton, MT USA; 4 Office of the Director, National Institute of Allergy and Infectious Diseases, National Institutes of Health, Bethesda, MD USA; 5 Biology of Vector-Borne Viruses Section, Laboratory of Virology, Rocky Mountain Laboratories, National Institute of Allergy and Infectious Diseases, National Institutes of Health, Hamilton, MT USA

**Keywords:** Microbiology, Molecular biology

## Abstract

Rocky Mountain spotted fever (RMSF) is a rapidly progressive and often fatal tick-borne disease caused by *Rickettsia rickettsii*. Its discovery and characterization by Howard Ricketts has been hailed as a remarkable historical example of detection and control of an emerging infectious disease, and subsequently led to the establishment of the Rocky Mountain Laboratories (RML). Here, we examined an unopened bottle of a vaccine, labeled as containing RMSF inactivated by phenol-formalin of infected ticks, developed prior to 1944 at RML by DNA analysis using Illumina high throughput sequencing technology. We found that it contains DNA from the Rocky Mountain wood tick (*Dermacentor andersoni*), the vector of RMSF, the complete genome of *Rickettsia rickettsii*, the pathogen of RMSF, as well as the complete genome of *Coxiella burnetii*, the pathogen of Q-fever. In addition to genomic reads of *Rickettsia rickettsii* and *Coxiella burnetii*, smaller percentages of the reads are from *Rickettsia rhipicephali* and *Arsenophonus nasoniae*, suggesting that the infected ticks used to prepare the vaccine carried more than one pathogen. Together, these findings suggest that this early vaccine was likely a bivalent vaccine for RMSF and Q-fever. This study is the among the first molecular level examinations of an historically important vaccine.

## Introduction

Rocky Mountain spotted fever (RMSF), was first recognized as an unknown disease called as “black measles” in the nineteenth century, especially after the 1890s in the Bitterroot Valley of southwest Montana^1^. Edward E Maxey^2,3^ provided the first clinical description of the so-called spotted fever of Idaho: “a febrile disease, characterized clinically by a continuous moderately high fever, and a profuse or purpuric eruption in the skin, appearing first on ankles, wrists, and forehead, but rapidly spreading to all parts of body”. The case fatality rate sometimes approached 80%^4^. The 1928 establishment and subsequent development of Rocky Mountain Laboratories (RML), component of the National Institutes of Health (NIH) since 1937, was a direct result of research on Rocky Mountain spotted fever that began around 1900, in the Bitterroot Valley^5^.

The causal agent of RMSF is a tick-borne rickettsial bacterium known as *Rickettsia rickettsii* (*R. rickettsii*), identified by pathologist Howard T. Ricketts^6^ and subsequently named in honor of his discovery^7^. The importance to medical history of this discovery was emphasized by Richard Shope who was presented with the Howard T. Ricketts Prize in 1963^8^. *Rickettsia rickettsii* is a gram-negative, intracellular, coccobacillus bacterium that is around 0.8 to 2.0 μm long^9^. After infection, its initial targets are CD68 + cells (macrophages and/or dendritic cells)^10^ which then spread hematogenously throughout the body and infect vascular endothelial cells. The bacteria can proliferate in the nucleus or in the cytoplasm of the infected host cell^11,12^. *Rickettsia rickettsii* possesses two major immunodominant surface proteins of outer membrane protein A (OmpA, 190 kDa) and outer membrane protein B (OmpB, 135 kDa), which is the most abundant surface protein of *Rickettsia*^3^. OmpA and OmpB contain species-specific epitopes that provide the basis for rickettsial serotyping in comparative indirect micro-immunofluorescence assays^13^. OmpA is important for *R. rickettsii* adhesion to host cells^14^ and interacts with α2β1 integrin to promote invasion of the bacteria into the host cells^15^. OmpB binds to host cell-specific receptor Ku70 (a subunit of a nuclear DNA-dependent protein kinase with subcellular localization in the cytoplasm and plasma membrane) and contributes to endocytosis and rickettsial internalization^16^. Both OmpA and OmpB are conserved throughout the spotted fever group *Rickettsia*, whereas OmpB is conserved in all *Rickettsia* species except for *R. canadensis*^17–19^. For this reason, some genetic studies have used the OmpA and OmpB gene region to distinguish different *Rickettsia* species^20–22^.

*Dermacentor andersoni* (*D. andersoni*), named in honor of John F. Anderson (1873–1958) in 1908^23^, is the primary vector of the RMSF (*R. rickettsii*)^24^, tularemia^25^, and Colorado tick fever (CTF) virus^26^. It also is the vector of Q fever^27^ and bovine anaplasmosis^28^. In 1903, John. F. Anderson (1873–1958) examined epidemiological data and found that all RMSF cases were associated with tick exposure in the week before the onset of spotted fever^29^. Adult *D. andersoni* feed on mammals, including humans, dog, horses, cattle, chipmunks, ground squirrels, marmots, and jackrabbits^30,31^. Its genome was sequenced by Agricultural Research Service, United States Department of Agriculture and submitted to GenBank in 2022 (GenBank Accession: JALBCO000000000).

In 1924, R. R. Spencer and R. R. Parker at RML prepared the first vaccine against RMSF by crushing infected ticks and phenol-inactivating the material^32^. In 1938, a simpler method of growing *Rickettsia* in the yolk sacs of developing chick embryo was developed by Herald R. Cox (1907–1986), utilizing formaldehyde inactivation, and extraction with ether^33^. Both vaccines showed protection in animal studies^34^ and were used in humans starting in 1927^35^, but neither vaccine conferred a high level of human immunity^36^. A subsequent RMSF vaccine developed in duck and chicken embryo culture followed by formalin inactivation had higher immunogenic activity and lower impurity^37,38^. However, complete protection against RMSF following vaccination with formalin-inactivated vaccines has not been achieved in humans to date, possibly because of alterations in the antigenic determinants due to the fixation method^39^. Recently, there have been studies of subunit^40,41^ or polypeptide^42^ vaccines of RMSF based on identified immunogenic surface proteins of *R. rickettsii*. Their studies showed that recombinant OmpA, OmpB, and Adr2 protein as antigens can develop antibody and T cell responses and provide protection in guinea-pigs^40^ and mice^41^. While Wang, et al.^42^ used immunodominant peptides as antigen in mice, which induce a Th1-type immune response against *R. rickettsii* infection. All these new studies and developments may lead to an effective new RMSF vaccine eventually. However, currently there is no licensed vaccine available for RMSF because of the effective treatment by doxycycline at early stage of infection^43^, decreased case fatality rate (from 28% in 1944 to 0.1% in 1995)^43^, and the limited understanding of the protective host response and the *R. rickettsii* antigens involved in stimulating protective immunity^44^.

Another intracellular bacterium, *Coxiella burnetii* (*C. burnetii*)*,* was identified as the causative agent of Q fever in the late 1930s. Q fever was first described by Derrick in abattoir workers in Brisbane, Queensland, Australia^45^. The pathogen of Q fever (*C. burnetii*) was discovered by Burnet (1899–1985) and Mavis Freeman when they studied one of Derrick’s patients in 1937^46^ and was near simultaneously isolated from *D. andersoni* at RML by Gordon David (1889–1977) and Herold Cox (1907–1986)^27^. *Coxiella burnetii*, named to honor both Cox and Burnet, is an obligate intracellular, small gram-negative bacterium (0.2 and 2.0 μm)^47^ with ~ 2 million base pair DNA genome^48^ and its phylogenic neighbors include Legionellae spp, Francisella tularensis, and Rickettsiella spp.^49^. When infected usually by inhalation of infectious aerosols generated by infected domestic livestock reservoirs such as dairy cows, goats, and sheep, Q fever generally presents in humans as an acute influenza-like illness followed by full recovery, particularly after treatment with doxycycline or other antibiotics^50^. However, because its high infectivity through aerosol route^51,52^, environmental resistance, and ability to cause disease, *C. burnetii* is classified as a Select Agent by the United States Centers for Disease Control and Prevention’s Division of Select Agents and Toxins (DSAT) and is a noted bioterrorism “pathogen of interest”^53^.

Q fever vaccine development started almost immediately following the identification of *C. burnetii* at RML, in infected ticks and later more-efficiently cultured in embryonated chicken eggs. Like the RMSF vaccine, this early Q-fever vaccine was made from formalin-inactivated infected *D. andersoni* tissues, and shown to offer protection in animal models^54^. The first Q fever vaccine introduced for use in humans, comprised of whole-cell, formaldehyde-inactivated, ether-extracted *C. burnetii* with 10% egg yolk sac^55^, and showed protection against high-dose aerosol challenge in US army volunteers^56^. The only currently available Q-fever vaccine, Q-vax, is an iteration of the whole cell vaccine from RML. Despite being different diseases with different epidemiology, bivalent RMSF and Q fever vaccines were made at RML, prepared from formalin-inactivated adult *D. andersoni* that were infected with agents for both Rocky Mountain spotted fever (*R. rickettsii*) and American Q fever (*C. burnetii*), and was protective in guinea pigs following challenge with both organisms^54^.

In the current study, an unopened 1944 vaccine bottle from RML labelled as “Rocky Mountain Spotted Fever Vaccine” was opened, DNA was extracted from the vaccine material and analyzed using Next Generation Sequencing (NGS) technology, which revealed the contents of the first RMSF vaccine produced in last century.

## Results

### DNA recovery

**Figure 1:**
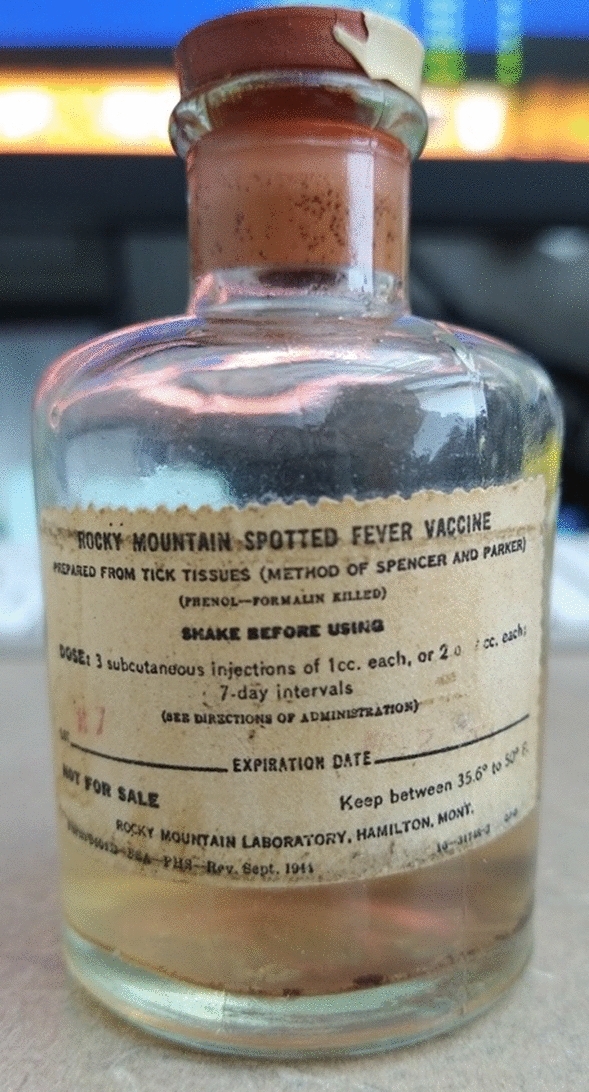
The bottle of Rocky Mountain Spotted Fever Vaccine made in 1944.

**Figure 2:**
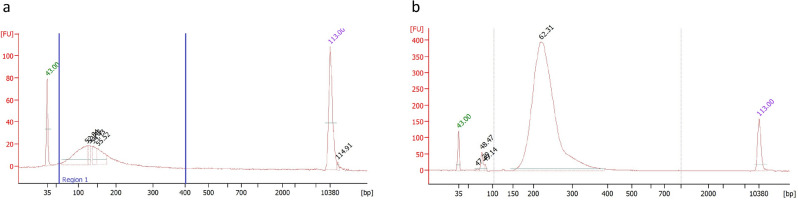
DNA size profile measured using a High Sensitivity DNA chip on an Agilent Bioanalyzer. (**a**) Isolated DNA from RMSF vaccine; (**b**) Final Illumina sequencing library with adaptor-ligated made from DNA isolated from RMSF vaccine. y-axis of the electropherograms represents fluorescent units (FU) and the x-axis represents the nucleotide length in base pair (bp).

An unopened, sealed glass bottle of Rocky Mountain Spotted Fever Vaccine was provided by Rocky Mountain Laboratories, National Institute of Health (Fig. 1). The manufacture date of this vaccine is September 1944. In this RMSF vaccine bottle, there were visible dark red small solid pieces floating in the liquid and settling at the bottom (Fig. 1). The pH value of the vaccine solution was 5.5. Test DNA isolations were performed on both solid and liquid portions from 400 µl of the vaccine contents. DNA was only recovered from the solid materials of the vaccine. The solid materials from a 3 ml vaccine suspension were collected from which DNA was isolated, yielding approximately 6 ng of DNA. The DNA size profile was in the range of 50-to-200 bp with a peak at ~ 120 bp (Fig. 2a).

### Illumina sequencing

Isolated DNA from RMSF vaccine solids was used to make an Illumina library without shearing since the peak DNA length was ~ 120 bp. The final constructed Illumina library profile is shown in Fig. 2b.

The constructed library was diluted and sequenced on a NextSeq 500 sequencer with a 160 bp single read run. At completion, a total of 83.9 Gb raw sequence data (616.45 M raw reads) with 82.3% of total bases >  = Phred score of Q30 was generated. All sequences generated were deposited as a series into the Genbank SRA database (Accession No. PRJNA880551).

### Sequence alignment to tick genome

As shown in Fig. 1, this RMSF vaccine was prepared from infected *D. andersoni* tick tissues. All generated Illumina reads were trimmed and aligned to reference genome of *Dermacentor andersoni* (JALBCO000000000.1) using Bowtie2^57^ with default settings. Among the total of 519,012,047 trimmed reads, 396,715,374 reads (76.44%) were aligned to the *D. andersoni* genome (with 203,363,636 (39.18%) aligned exactly 1 time and 193,351,738 (37.25%) aligned > 1 times). The average genome coverage for all 3120 contigs of *D. andersoni* genome was 94.6%. Therefore, DNA sequence analysis confirmed that this vaccine was prepared from infected *D. andersoni* ticks.

### Sequence alignment to rickettisal genomes

To confirm the presence of rickettisal species DNA in this vaccine, all sequences were aligned to 14 complete rickettisal genomes that downloaded from NCBI. The numbers of aligned reads and percentages are shown in Table 1: the species with the most aligned reads was *R. rickettsii*. The average of genome coverage of *R. rickettsii* is 1021.58 times and 99.7% of the genome (1,268,201 bp) is covered by the mapped reads (Table 2).

**Table 1:** Aligned reads to complete rickettisal genomes.

Rickettisal species	GenBank accession	Total reads	Aligned onetime reads	Aligned > 1 time reads	Total aligned reads	Overall alignment rate
*R. akari*	GCF_000018205.1	519,012,047	5,682,463	6631	5,689,094	1.10%
*R. asiatica*	GCF_007989425.1	519,012,047	7,149,491	38,500	7,187,991	1.38%
*R. australis*	GCF_000284155.1	519,012,047	6,295,260	16,953	6,312,213	1.22%
*R. bellii*	GCF_002078315.1	519,012,047	1,695,792	49,782	1,745,574	0.34%
*R_canadensis*	GCF_000283915.1	519,012,047	3,892,560	796	3,893,356	0.75%
*R. conorii*	GCF_000007025.1	519,012,047	10,296,428	54,786	10,351,214	1.99%
*R. monacensis*	GCF_000499665.2	519,012,047	7,336,041	58,005	7,394,046	1.42%
*R. prowazekii*	GCF_000277165.1	519,012,047	2,686,599	1268	2,687,867	0.52%
*R. rhipicephali*	GCF_000284075.1	519,012,047	10,322,074	62,102	10,384,176	2.00%
*R. rickettsii*	GCF_000017445.4	519,012,047	10,548,147	56,711	10,604,858	2.04%
*R. slovaca*	GCF_000237845.1	519,012,047	10,408,576	61,641	10,470,217	2.02%
*R. sp.MEAM1*	GCF_002285905.1	519,012,047	1,216,894	29,885	1,246,779	0.24%
*R. tillamookensis*	GCF_016743795.1	519,012,047	6,746,940	69,293	6,816,233	1.31%
*R. typhi*	GCF_000277285.1	519,012,047	2,596,733	1170	2,597,903	0.50%

**Table 2:** Alignment results of *R. rickettsii* genome.

*R. rickettsii* genome	Genome length	Mapped reads	Genome covered length	Genome covered rate	Genome average coverage
NC_010263.3	1,268,201	10,604,858	1,264,873	0.9973758	1021.58

There were a total of 27,909 single nucleotide polymorphisms (SNPs) observed compared to the reference genome (see Materials and Methods) with 11,790 nonsynonymous SNPs (Supplemental Table 1). The consensus *R. rickettsii* vaccine-derived sequence was submitted to GenBank with accession number of CP114277.

As shown in Table 1, *R. rickettsii* has the most aligned reads (2.04%), but *R. slovaca* (2.02%), *R. rhipicephali* (2.0%), and *R. conorii* (1.99%) also had significant aligned reads. All of them belong to the rickettsial spotted fever group and their phylogenetic relationships are close to each other^58^. Previous studies have used OmpA, OmpB, and GltA genes to distinguish different rickettsial species^20–22,59^. Therefore, the obtained SNPs at OmpA gene region (5,855 bp, from 1,176,559 to 1,182,413 of NC_010263.3), OmpB gene region (4,962 bp, from 1,014,075 to 1,019,036 of NC_010263.3), and GltA gene region (1,305 bp, from 1,212,499 to 1,213,803 of NC_010263.3) from *R. rickettsii* were compared to the differences at these regions of *R. slovaca*, *R. rhipicephali*, and *R. conorii* genomes respectively (Supplemental Table 2).

A total of 221 SNPs were identified in the OmpA region from the vaccine-derived DNA library. Among them, there are 12 called SNPs that have variant base numbers in which > 50% of total aligned read numbers and the remaining SNPs (209) are all minor SNPs with their variant rates ranging from 10.26% to 49.28%. Therefore, there are only 12 SNP differences from the vaccine-derived consensus OmpA gene sequence as compared to the reference *R. rickettsii* (NC_010263.3) sequence, while at all these 221 called SNP sites, 175 of them are different bases between reference genomes of *R. rickettsii* and *R. rhipicephali*; 64 are different between *R. rickettsii* and *R. slovaca*; and 71 are different between *R. rickettsii* and *R. conorii* (Table 3, Supplemental Table 2).

**Table 3:** Results of SNP analysis at OmpA, OmpB, and GltA gene regions.

	OmpA	OmpB	GltA
Total called SNP number compared to *R. rickettsii* reference	221	207	21
Bases are different between *R. rickettsii* and *R. rhipicephali*	175	200	15
Bases are different between *R. rickettsii* and *R. slovaca*	64	72	5
Bases are different between *R. rickettsii* and *R. conorii*	71	73	6
SNP matching *R. rhipicephali*	167	199	15
SNP matching *R. rhipicephali* only	109	126	10
SNP matching *R. slovaca*	61	66	5
SNP matching *R. slovaca* only	9	0	0
SNP matching *R. conorii*	62	66	6
SNP matching *R. conorii* only	2	0	1

A total of 207 SNPs were identified in the OmpB region from the vaccine-derived DNA library. Among them, only 4 have variant base numbers in which > 50% of total aligned read numbers were observed at these positions and the remaining SNPs (203) are all minor SNPs with their variant rates ranging from 10.64% to 38.05%. Therefore, there are only 4 SNP differences from the vaccine-derived consensus OmpB gene sequence as compared to the reference *R. rickettsii* (NC_010263.3) sequence, while at all these 207 called SNP sites, 200 of them are different between reference genomes of *R. rickettsii* and *R. rhipicephali*; 72 are different between *R. rickettsii* and *R. slovaca*; and 73 are different between *R. rickettsii* and *R. conorii* (Table 3, Supplemental Table 2).

A total of 21 SNPs were identified in the GltA region from the vaccine-derived DNA library and none of them has variant base numbers in which > 50% of total aligned read numbers. So all called SNPs at GltA gene region are all minor SNPs with their variant rates ranging from 14.40% to 31.99%. Therefore, there are no SNP differences from the vaccine-derived consensus GltA gene sequence as compared to the reference *R. rickettsii* (NC_010263.3) sequence, while all these 21 called SNP sites, 15 of them are different between reference genomes of *R. rickettsii* and *R. rhipicephali*; 5 are different between *R. rickettsii* and *R. slovaca*; and 6 are different between *R. rickettsii* and *R. conorii* (Table 3, Supplemental Table 2).

Hence, after analysis of called SNPs at OmpA, OmpB, GltA gene regions, the obtained consensus *Rickettsia* sequence is *R. rickettsii* as expected according to the method labeled on the vaccine bottle and used in RML vaccine preparations^32^.

Among these compared 175 SNP sites in OmpA region that are different between *R. rickettsii* and *R. rhipicephali*, 167 of called SNP bases based on our obtained sequences are the same as the bases in *R. rhipicephali* and 109 of them are unique to *R. rhipicephali*. Among these compared 64 sites in OmpA region that are different between *R. rickettsii* and *R. slovaca*, 61 of called SNP bases are the same as the bases in *R. slovaca* and only 9 of them are unique to *R. slovaca*. Among these compared 71 sites in OmpA region that are different between *R. rickettsii* and *R. conorii*, 62 of called SNP bases are the same as the bases in *R. conorii,* but only 2 of them are unique to *R. conorii* (Table 3, Supplemental Table 2).

Among these compared 200 SNP sites in OmpB region that are different between *R. rickettsii* and *R. rhipicephali*, 199 of called SNP bases based on our obtained sequences are the same as the bases in *R. rhipicephali* and 126 of them are unique to *R. rhipicephali*. Among these compared 72 sites in OmpB region that are different between *R. rickettsii* and *R. slovaca*, 66 of called SNP bases are the same as the bases in *R. slovaca*, but none of them are unique to *R. slovaca*. Among these compared 73 sites in OmpB region that are different between *R. rickettsii* and *R. conorii*, 66 of called SNP bases are the same as the bases in *R. conorii*, none of them are unique to *R. conorii* either (Table 3, Supplemental Table 2).

Among these compared 15 SNP sites in GltA region that are different between *R. rickettsii* and *R. rhipicephali*, all 15 of called SNP bases based on our obtained sequences are the same as the bases in *R. rhipicephali* and 10 of them are unique to *R. rhipicephali*. Among these compared 5 sites in GltA region that are different between *R. rickettsii* and *R. slovaca*, 5 of called SNP bases are the same as the bases in *R. slovaca*, but none of them are unique to *R. slovaca*. Among these compared 6 sites in GltA region that are different between *R. rickettsii* and *R. conorii*, 6 of called SNP bases are the same as the bases in *R. conorii*, but only 1 of them are unique to *R. conorii* (Table 3, Supplemental Table 2).

Therefore, after analysis of called SNPs at OmpA, OmpB, GltA gene regions with base differences among *R. rickettsii, R. rhipicephali, R. slovaca,* and *R. conorii*, this RMSF vaccine likely contains at least 10% of *R. rhipicephali*.

### Bacterial metagenomic analysis

**Figure 3:**
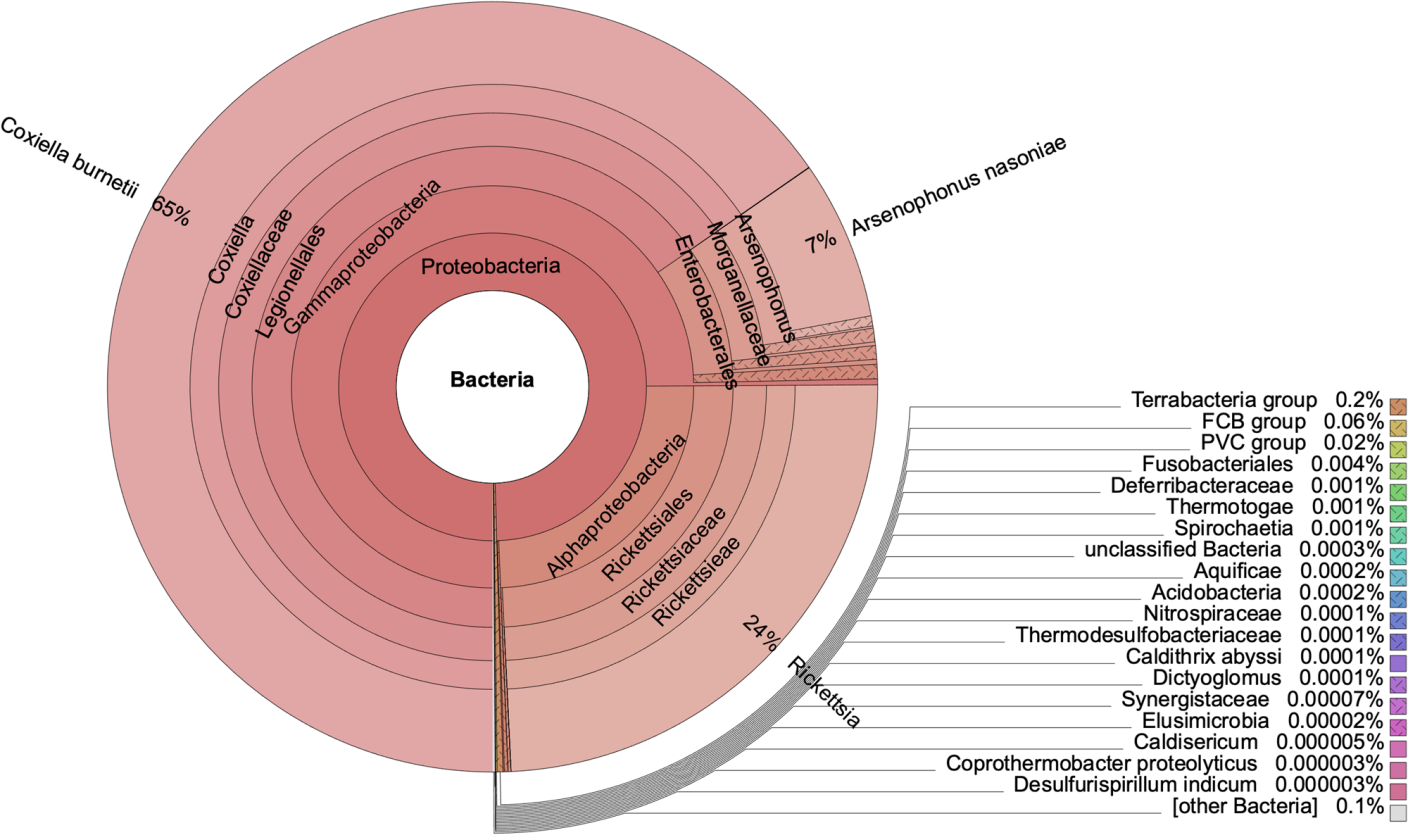
Metagenomics analysis of bacterial group in sequenced RMSF vaccine.

Bacterial metagenomic analysis was performed on trimmed, filtered and collapsed reads using Kraken2^60^ and the result is shown in Fig. 3. It not only confirms that about 24% of the reads align to rickettisal genomes, but 65% of reads aligned to the *C. burnetii* genome, and 7% of the reads aligned to the *Arsenophonus nasoniae* (*A. nasoniae*) genome that is also carried by ticks^61,62^. *Arsenophonus nasoniae* is a gram-negative gammaproteobacterial, secondary-endosymbiont that infects a wide range of insects and arachnids^63^.

### Sequence alignment to *Coxiella burnetii*

Because metagenomic analysis showed large portion of the bacterial reads belong to *C. burnetii*, all generated Illumina reads were trimmed and aligned to reference genome of *C. burnetii* RSA 493 (PRJNA57631) using Bowtie2^57^. Among the total of 519,012,047 trimmed reads, 35,411,467 reads (6.86%) were aligned to the *C. burnetii* genome (34,704,131 (6.69%) aligned exactly 1 time and 707,336 (0.14%) aligned > 1 times), which covered the *C. burnetii* genome and its plasmid with 100% with an average genome coverage of 1954 times (Table 4). Therefore, this RMSF vaccine also contains whole genomes of *C. burnetii*. Compared to the reference *C. burnetii* genome RSA 493 (PRJNA57631), there are a total of 273 SNPs (268 in the chromosome and 5 in the plasmid) identified SNPs (Materials and methods) with only 7 nonsynonymous changes (Supplemental Table 3). The consensus sequence of this obtained *C. burnetii* was submitted to GenBank with accession number of CP115461.

**Table 4:** Alignment results of *C. burnetii* genome.

*C. burnetii* genome	Type	Genome length	Mapped reads	Genome covered length	Genome covered rate	Genome average coverage
NC_002971.4	Chromosome	1,995,488	34,776,864	1,995,488	1.0000000	1954
NC_004704.2	Plasmid	37,319	634,603	37,319	1.0000000	1954

### Sequence alignment to *Arsenophonus nasoniae*

Another bacterial group with significant match identified by Kracken2 was *Arsenophonus nasoniae* (*A. nasoniae*). All generated Illumina reads were trimmed and aligned to reference genome of *A. nasoniae* (PRJNA529362). Among the total of 519,012,047 trimmed reads, only 3,590,436 reads (0.69%) were aligned to *A. nasoniae* genome (3,297,383 (0.64%) aligned exactly 1 time and 293,053 (0.14%) aligned > 1 times), with a 32.9% coverage of the *A. nasoniae* genome with chromosome coverage of 40.8% (Table 5). This confirms that this RMSF vaccine contained a low level of *A. nasoniae* DNA.

**Table 5:** Alignment results of *A. nasoniae* genome.

*A. nasoniae*	Type	Genome length	Mapped reads	Genome covered length	Genome covered rate
NZ_CP038613.1	Chromosome	3,871,978	3,389,947	1,578,353	0.4076348
NZ_CP038614.1	Plasmid	222,851	85,105	19,898	0.0892884
NZ_CP038615.1	Plasmid	85,190	3341	2591	0.0304144
NZ_CP038616.1	Plasmid	85,274	6085	2486	0.0291531
NZ_CP038617.1	Plasmid	133,306	14,249	6201	0.046517
NZ_CP038618.1	Plasmid	61,872	14,176	4934	0.0797453
NZ_CP038619.1	Plasmid	121,472	4817	4282	0.0352509
NZ_CP038620.1	Plasmid	120,926	23,469	6750	0.0558193
NZ_CP038621.1	Plasmid	51,789	1602	1311	0.0253143
NZ_CP038622.1	Plasmid	50,328	1590	1261	0.0250556
NZ_CP038623.1	Plasmid	46,250	2742	1526	0.0329946
NZ_CP038624.1	Plasmid	34,725	34,938	5886	0.1695032
NZ_CP038625.1	Plasmid	33,626	8132	6648	0.1977042
NZ_CP038626.1	Plasmid	32,417	2	105	0.003239
NZ_CP038627.1	Plasmid	15,977	0	0	0
NZ_CP038628.1	Plasmid	8312	3	115	0.0138354
NZ_CP038629.1	Plasmid	3641	62	76	0.0208734
NZ_CP038630.1	Plasmid	7173	176	503	0.0701241

## Discussion

As shown on the label of the bottle, the 1944 vaccine was prepared from phenol-formalin inactivated infected tick tissues using the method of Spencer and Parker^32^ (Fig. 1). In the vaccine bottle, pieces of dark red material, as large as 2 mm squared, were visible floating in the solution and settling at the bottom. According to the original method, the precipitate that formed after adding phenol was separated by slow centrifugation and only the supernatant was used as a vaccine, which had a moderate turbidity^32^. Therefore, the dark red material that is visible in the vaccine currently probably represents tick tissue re-precipitation after 78 years. In our study, DNA was recovered only from the solid materials in the vaccine, not the solute, which also may help explain that the potency of the original vaccine was destroyed if passed through a Berkefeld filter^32^. In addition, because the final vaccine process is adding phenol to kill extraneous organisms, the pH value of the vaccine solution of 5.5 is explicable.

The first generation RMSF vaccine was made from crushed Rocky Mountain wood ticks (*D. andersoni*) that fed on *R. rickettsii-*infected guinea pigs^32^. Therefore, sequences from *D. andersoni* and *R. rickettsii* were expected and our sequence results confirm this. However, the unexpected result is that, within the bacterial metagnomic analysis, the bacterial genome with the most reads was not *Rickettsia*, but *C. burnetii*, the pathogen causing Q fever^27^. In 1940 at RML, Cox and John E. Bell prepared 10 vaccines from adult *D. andersoni* that were infected with both Rocky Mountain spotted fever and Q fever pathogens. They showed complete protection of guinea pigs against at least 1000 infectious doses of spotted fever and against at least 10,000 doses of Q fever rickettsiae (the genus name *Coxiella* was not established at that time)^54^. Although the label of this bottle of vaccine sequenced here does not mention Q fever, this vaccine is thus possibly one of these bivalent vaccines prepared from ticks harboring the two infectious agents simultaneously^54^. It seems reasonable that a vaccine against both agents would be useful to laboratory personnel working with these bacteria at that time since RMSF was often a fatal disease and Q fever was highly infectious in laboratory settings.

Low levels of *R. rhipicephali* DNA were also identified in this vaccine. Since *R. rhipicephali* was first isolated from the brown dog tick (*Rhipicephalus sanguineus*) in Mississippi^64^, it has been isolated in diverse tick genera (*Haemaphysalis juxtakochi*, *Ixodes ricinus*, *D. occidentalis*, *D. andersoni*, and *D. variabilis*) with wide geographic distribution^65–67^. Although *R. rhipicephali* belongs to the spotted fever group *Rickettsia* as does *R. rickettsii*, it has not been identified as a human pathogen, only showing moderately severe disease in meadow voles inoculated with it^68^. However, at least six of subspecies of spotted fever rickettsiae have been isolated from ticks, and later found to be pathogenic to humans^69^. The first mixed infection of *R. belli, R. montanensis,* and *R. rickettsii* in one tick was reported in 2006^70^. However, no ticks were found to be co-infected with *R*. *rhipicephali* and another spotted fever group *Rickettsia*^71^. In the current study, DNA sequence analysis suggests a mixed infection of *R. rickettsii* and *R. rhipicephali* in the infected ticks from which the vaccine was prepared. However, the possibility that different ticks infected with *R. rickettsii* and *R. rhipicephali* respectively were mixed in the vaccine preparation cannot be ruled out. In addition, the SNPs reported in Supplemental Table 1, especially the ones at low levels, probably reflect the mixture of *R. rickettsii* and *R. rhipicephali* in the RMSF vaccine.

We identified the bacterium *A. nasoniae* in this vaccine, *which* was first isolated from the parasitic wasp *Nasonia vitripennis* and is the causative agent of the son-killer trait in that species^72^. *Arsenophonus nasoniae* is a maternally-inherited parasitic bacterium that can cause lethality in approximately 80% of male embryos produced by infected female wasps^73^. *Arsenophonus nasoniae* has been found in wide range of insects and arachnids^74^, including ticks (*Dermacentor andersoni*, *Dermacentor variabilis, and Ixodes ricinus*)^62,75,76^. Therefore, its presence in our study is not unexpected.

Lastly, avoiding DNA contamination during the whole process of the experiments was critical. Handling of the vaccine vial and processing the samples were always performed in biological safety cabinets. In addition, the laboratory where the extraction and sequencing occurred and laboratories in the same building have never conducted prior research on these bacteria. The lowest obtained reads that we reported in the RMSF vaccine has more than 3.3 million from *A. nasoniae*, which is highly unlikely from the material introduced during our experimental handling of the vaccine.

## Conclusions

Recent studies of an historical 1902 vaccine used against smallpox have likewise revealed unexpected DNA results, e.g., that it did not contain the vaccinia virus, as expected, but the horsepox virus instead^77,78^. Here, we have documented the history of another important vaccine by revealing that a 1944 RMSF vaccine prepared at RML contained *D. andersoni* and *R. rickettsii* DNA, which confirms its preparation from *R. rickettsii*-infected Rocky Mountain Wood ticks (*D. andersoni*). However, our work also reveals that it included significant amounts of *C. burnetii* DNA and low amount of *R. rhipicephali* DNA, which suggests it was a bivalent RMSF and Q-fever vaccine produced from ticks with a mixed infection of both *R. rickettsii* and *R. rhipicephali*. This is the first study applying modern high throughput sequencing technology to investigate an early RMSF vaccine made at RML and is thus represents an invaluable piece of history for the Rocky Mountain Laboratories and the National Institutes of Health.

## Materials and methods

### DNA isolation

Test DNA isolation was performed on 400ul of well-shaken RMSF vaccine solution. Liquid and solid portions of the vaccine solution were obtained by centrifuge at 13,000 rpm at 4 °C for 10 min. DNA isolation from the liquid portion was performed using NucleoSpin cfDNA isolation kit from Takara Bio (San Jose, CA) following manufacturer’s instructions. DNA isolation from the solid portion was performed using Zymo Quick-DNA™ Miniprep Plus Kit from Zymo Research (Irvine, CA) following manufacturer’s instructions. Final volumes of isolated DNA were 10 µl and 1 µl from each isolation, and they were measured using the Agilent High Sensitivity DNA Kit (Agilent Technologies, Santa Clara, CA) and Qubit (Thermo Fisher Scientific, Waltham, MA). The DNA concentration from solid portion was 0.05–0.1 ng/µl and no DNA could be detected from the isolation of liquid portion by either method. Therefore, after centrifuging 2 × 1.5 ml vaccine solution at 13,000 rpm at 4 °C for 10 min, supernatants were put back into original RMSF vaccine bottle and precipitations were used to isolated DNA using Zymo Quick-DNA™ Miniprep Plus Kit. From this larger preparation, about 6 ng DNA was obtained for Illumina library construction.

### Library construction and sequencing

Illumina sequencing library was made from isolated DNA by using NEBNext® Ultra™ II DNA Library Prep Kit from New England Biolabs (Ipswich, MA) following manufacture instructions. The final sequencing library was analyzed with the Agilent 2100 Bioanalyzer using the Agilent High Sensitivity DNA Kit (Agilent Technologies, Santa Clara, CA) (Fig. 2). The constructed library was diluted following Illumina sequencing standard protocols and sequenced on a NextSeq 500 sequencer with 160 bp single read run using NextSeq 500 High Output Kit v2.5 (Illumina, San Diego, CA).

### Data analysis

Illumina reads were trimmed using Trimmomatic^79^ and mapped to reference genomes including *Dermacentor andersoni* (JALBCO000000000.1 and the access date is 07/28/2022 ), *Coxiella burnetii* (PRJNA57631 and the access date is 05/09/2022), *Arsenophonus nasoniae* (PRJNA529362 and the access date is 05/16/2022), and 14 complete *Rickettsia* genomes (GenBank accession numbers are in Table 1 and access date is 08/05/2022) that downloaded from NCBI using Bowtie2 (version 2.3.4.1) with default settings^57^. *Rickettsia rickettsii* consensus sequence was generated based on reference *R. rickettsii* (CP000848.1). SAMtools mpileup (version 2.1.0)^80^ was used to generate mpileup files that were subsequently used to make SNP calls by VarScan2^81^. Bacterial genome classification in all the samples was performed using Kraken2^60^, utilizing its standard database on the trimmed using Trimmomatic with default settings^79^ , filtered using FASTX-Toolkit (http://hannonlab.cshl.edu/fastx_toolkit/) with -q 20 -p 80, and collapsed to remove duplicates using FASTX-Toolkit (http://hannonlab.cshl.edu/fastx_toolkit/) reads. A reported SNP call using VarScan2^81^ was the one that satisfied the following criteria at the SNP position: (1) more than 100 reads at that position, (2) minimum base Phred quality score as 25, (3) the different bases were more than 10% of the aligned reads, (4) pass VarScan2 Strand Filter, (5) VarScan2 SNP call p-value less than 0.05. Consensus nucleotide sequence was generated by VarScan2 with minimum coverage of 100 reads, minimum average quality value of 25, minimum variant frequency 0.5, and SNP call p-value less than 0.05.

## Supplementary Information

Supplementary Information 1.

Supplementary Information 2.

Supplementary Information 3.

## Data Availability

The datasets generated and/or analyzed during the current study are available in the NCBI SRA database with accession numbers: PRJNA880551, CP114277, and CP115461.
